# Alliance for Sleep Clinical Practice Guideline on Switching or Deprescribing Hypnotic Medications for Insomnia

**DOI:** 10.3390/jcm12072493

**Published:** 2023-03-25

**Authors:** Nathaniel F. Watson, Ruth M. Benca, Andrew D. Krystal, William V. McCall, David N. Neubauer

**Affiliations:** 1 Department of Neurology, University of Washington School of Medicine, Seattle, WA 98195, USA; 2 Department of Psychiatry and Behavioral Medicine, Wake Forest School of Medicine, Winston-Salem, NC 27101, USA; 3 Department of Psychiatry and Neurology, UCSF Weill Institute for Neurosciences, San Francisco, CA 94158, USA; 4 Department of Psychiatry and Health Behavior, Medical College of Georgia, Augusta University, Augusta, GA 30912, USA; 5 Department of Psychiatry and Behavioral Sciences, School of Medicine, Johns Hopkins University, Baltimore, MD 21218, USA

**Keywords:** insomnia, insomnia medications, hypnotics, switching, deprescribing

## Abstract

Determining the most effective insomnia medication for patients may require therapeutic trials of different medications. In addition, medication side effects, interactions with co-administered medications, and declining therapeutic efficacy can necessitate switching between different insomnia medications or deprescribing altogether. Currently, little guidance exists regarding the safest and most effective way to transition from one medication to another. Thus, we developed evidence-based guidelines to inform clinicians regarding best practices when deprescribing or transitioning between insomnia medications. Five U.S.-based sleep experts reviewed the literature involving insomnia medication deprescribing, tapering, and switching and rated the quality of evidence. They used this evidence to generate recommendations through discussion and consensus. When switching or discontinuing insomnia medications, we recommend benzodiazepine hypnotic drugs be tapered while additional CBT-I is provided. For Z-drugs zolpidem and eszopiclone (and not zaleplon), especially when prescribed at supratherapeutic doses, tapering is recommended with a 1–2-day delay in administration of the next insomnia therapy when applicable. There is no need to taper DORAs, doxepin, and ramelteon. Lastly, off-label antidepressants and antipsychotics used to treat insomnia should be gradually reduced when discontinuing. In general, offering individuals a rationale for deprescribing or switching and involving them in the decision-making process can facilitate the change and enhance treatment success.

## 1. Introduction

Insomnia, defined as difficulty falling or staying asleep or early morning awakening, resulting in some form of daytime impairment, is the most common sleep disorder in adults, with a worldwide prevalence of 10–30% [1,2,3]. Sleep affects nearly every aspect of human physiology, and when compromised by insomnia, it can have broad untoward effects on human health, functioning, and quality of life [4]. Daytime impairments in cognitive functioning and alertness can compromise work performance and increase the risk of motor vehicle crash [5,6].

The causes of insomnia are diverse [7]. Comorbid sleep or medical disorders, such as obstructive sleep apnea, chronic pain, or depression and anxiety, can contribute to insomnia incidence, and although treatment of the comorbidity can improve insomnia symptoms, the insomnia should also be treated independently to optimize therapeutic outcomes [8,9,10,11,12]. Polypharmacy for comorbidities can also contribute to insomnia, depending on the specific medications in question, due to untoward side effects or medication interactions [13]. Thus, insomnia management is complex.

Cognitive behavioral therapy for insomnia (CBT-I), in which individuals with insomnia are provided with techniques for managing their insomnia non-medicinally, is a proven treatment, although access to this therapeutic modality is limited [14]. As such, many clinicians and their patients turn to insomnia-focused medicinal therapies in search of insomnia relief. The major categories of drugs approved by the U.S. Food and Drug Administration (FDA) for the treatment of insomnia include prescription hypnotic drugs such as benzodiazepines (BZDs; estazolam, flurazepam, quazepam, temazepam, triazolam), “Z-drugs” (eszopiclone, zaleplon, zolpidem), dual orexin receptor antagonists (DORAs; daridorexant, lemborexant, suvorexant), melatonin receptor agonists (ramelteon), and low doses of the tricyclic antidepressant doxepin. Clinicians also frequently prescribe medications not specifically indicated for insomnia (e.g., BZDs, antidepressants, antipsychotics, and anticonvulsants) to relieve symptoms. Indeed, the frequency of medication use for insomnia is increasing, with an estimated 9 million adults in the U.S. reporting prescription medication use for sleep disturbance [15,16,17,18]. 

Insomnia medications are typically time limited in their use; however, for many, the chronic nature of insomnia necessitates long-term treatment with medications. Since any given insomnia medication will not be effective for everyone, multiple medication trials may be necessary to find the optimum treatment. Similarly, issues such as medication side effects, untoward interactions with co-administered medications, and loss of therapeutic effect over time resulting in enduring insomnia symptoms can necessitate switching between different insomnia medications (often from one category of medication to another) or deprescribing altogether. In fact, deprescribing benzodiazepine receptor agonists (BzRAs) used for insomnia is advocated by multiple clinical practice guidelines and professional medical societies, particularly in older individuals [1,19,20,21,22]. Despite this clinical reality, clinicians have little guidance regarding the most effective and safest way to make these medication switches or discontinuations. Potential problematic issues during these transitions include rebound insomnia and corresponding symptom exacerbation, withdrawal symptoms, seizure precipitation, and impacts on the perception of efficacy of the next medication trialed. Rebound insomnia promotes anticipatory anxiety around sleep and can reinforce the notion that the individual cannot sleep without medication. 

The purpose, therefore, of this clinical practice guideline is to review the evidence surrounding insomnia medication transitions and deprescribing to identify the best means of discontinuing each type of medication therapy and of switching among the different treatment options. Following the evidence review, a group of five sleep medicine experts rated the quality of evidence and used the evidence to generate clinical recommendations for how best to deprescribe or discontinue insomnia medications to maximize safety and support optimal therapeutic outcomes.

## 2. Materials and Methods

A PUBMED search was carried out to identify studies addressing the effects of or optimal ways of switching, deprescribing, or tapering insomnia medications. This is a systematic review [23]. Literature searches were performed in December 2022 for each insomnia drug class, and searches were limited to human research studies in the English language. We focused on the last 10 years, when publication volume was extensive. The search terms included ‘switching AND insomnia’, ‘deprescribing AND insomnia’, ‘tapering AND insomnia’, or ‘discontinuation AND insomnia’ AND each compound name (e.g., eszopiclone, trazodone) or each of the drug class names (e.g., nonbenzodiazepine sedative hypnotics, DORAs, etc.) AND the terms ‘withdrawal’ and ‘rebound’. Additional articles were retrieved from citations of the articles identified in the initial search and included if eligible. 

For the benzodiazepine drug group, the following drug-specific terms were used: benzodiazepine, hypnotics, estazolam, flurazepam, temazepam, quazepam, and triazolam. From these results, 123 BZD-drug-related articles were reviewed, and 31 articles were considered relevant and selected for presentation and discussion.

For the Z-drug group, the following drug-specific terms were used: nonbenzodiazepine sedative hypnotics, benzodiazepine-like hypnotics, eszopiclone, zaleplon, zolpidem, and Z-drugs. From these results, 80 Z-drug-related articles were reviewed, and 28 articles were considered relevant and selected for presentation and discussion.

For the DORA+ Doxepin/Ramelteon drug group, the following drug-specific terms were used: DORAs, dual orexin antagonists, suvorexant, lemborexant, daridorexant, doxepin, and ramelteon. From these results, 95 drug-related articles were reviewed, and 12 articles were considered relevant and selected for presentation and discussion.

For the “Off-label” drug group, the following drug-specific terms were used: trazodone, quetiapine, mirtazapine, amitriptyline, imipramine, and tricyclic antidepressants. From these results, 82 off-label drug-related articles were reviewed, and 28 articles were considered relevant and selected for presentation and discussion.

**Figure 1:**
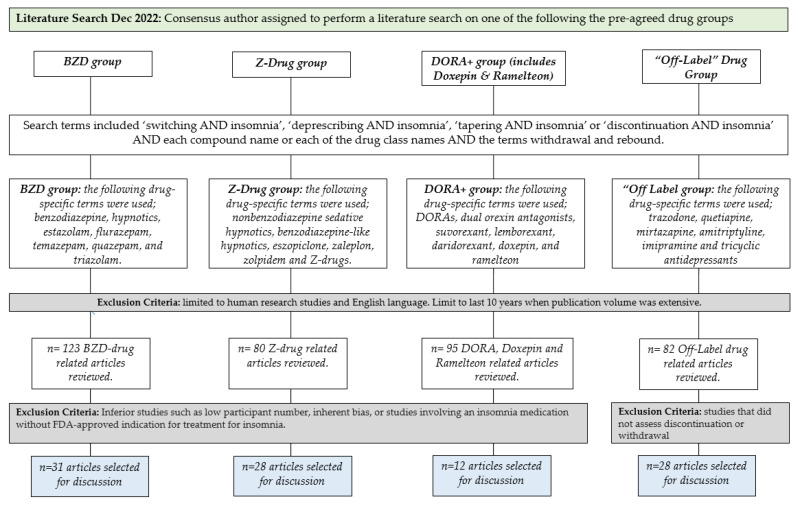
Flow chart summarizing the methodological approach utilized to generate reommendations for best practices during insomnia medication transitions and medication deprescribing.

A literature search flow chart outlining the procedure taken for each drug class can be seen in Figure 1 below:

Prior to summarization of the selected studies, the panel agreed on a grading-of-evidence scale. Once each sleep specialist had carried out their evidence-based review for their assigned insomnia drug treatment group, they graded the literature as follows:

A—evidence obtained from meta-analysis, including at least one large, randomized control trial (RCT).

B—evidence obtained from either meta-analysis, including at least one small RCT, or from at least one well-designed large RCT.

C—evidence obtained from well-designed cohort or case-controlled studies.

D—evidence obtained from case series, case reports, or flawed clinical trials. 

E—opinions of respected authorities based on clinical experience, descriptive studies, or reports of expert committees. 

Where insufficient evidence was available in the literature, the group discussed the topic, and opinions were shared and recorded. Thus, this clinical guideline includes both evidence-based and consensus-based recommendations. Issues such as the medication’s specific mechanism of action, dose, length of prescription, comorbidities, and medication pharmacology (e.g., elimination half-life) were all considered in the process of generating this clinical practice guideline. 

## 3. Results

### 3.1. Literature Review

#### 3.1.1. BZD Drug Discontinuation 

This section summarizes the literature surrounding the discontinuation of the following benzodiazepine hypnotics (a.k.a. GABA-A positive allosteric modulators): estazolam, flurazepam, temazepam, quazepam, and triazolam. Literature investigating the risk of withdrawal and likelihood of rebound insomnia upon discontinuation was prioritized (Table 1).

#### 3.1.2. Risk of Adverse Effects of BZD Drug Discontinuation

Abrupt discontinuation of BZDs can lead to both withdrawal and rebound insomnia [24]. There is a higher risk for rebound insomnia associated with drugs with shorter half-lives (for example, triazolam) versus those with longer half-lives; risk also escalates when drugs are taken at higher versus lower doses [25,26,27]. Rebound insomnia following abrupt discontinuation of BZDs can last for several days and may be accompanied by increased anxiety. Daytime anxiety and terminal or “late” insomnia have been reported in individuals using triazolam, particularly at high doses, suggestive of a withdrawal reaction mere hours after taking the medication [28,29]. Withdrawal symptoms and rebound insomnia from discontinuation of short-acting BZDs generally occur within 1–2 days, whereas the onset of withdrawal symptoms and rebound insomnia (if present) may not start until 2–7 days after stopping long-acting drugs [30]. Though severe withdrawal reactions (e.g., seizures) have rarely been reported following discontinuation of therapeutic doses, this could be a concern for individuals who are taking high doses of BZDs, or those who are taking BZDs during the daytime (e.g., for anxiety) as well as for sleep, and then abruptly stop medication.

Several studies have assessed switching from BZDs to other classes of hypnotics [31,32]. A retrospective study assessed the effects of either adding on suvorexant to a BZD vs. switching from a BZD to suvorexant in 228 subjects who were taking a variety of BZD or non-benzodiazepine receptor agonists at an average diazepam equivalent dose of 8.5 + 5.8 mg [31]. In the add-on group, researchers found suvorexant led to more oversedation and a significantly higher rate of suvorexant discontinuation due to intolerability than in the switch group [31]. In another retrospective report, individuals with insomnia were offered an opportunity to switch from the BZD diazepam (equivalent dose 4.6 + 2.8 mg) to lemborexant, either by adding on lemborexant prior to or while tapering the BZD, or by switching to lemborexant directly [32]. Of 180 subjects, 57/137 (42%) chose not to switch from the BZD; these individuals were on significantly higher BZD doses for a longer duration than those who elected to switch. Subjects (*n* = 80) who were switched from a BZD to lemborexant were able to do so with minimal adverse effects and a concomitant reduction in the Athens Insomnia Scale score. Thus, longer duration of treatment with BZDs and higher doses were associated with greater difficulty switching to lemborexant [32].

In an open-label, randomized study, 18 subjects with comorbid insomnia and major depressive disorder were investigated. Participants were currently taking various benzodiazepine hypnotics for at least 2 weeks but had residual insomnia (Insomnia Severity Index (ISI) score > 7) [33]. Subjects were randomized to receive either suvorexant or eszopiclone, and benzodiazepine dosage was reduced by half during the first 2 weeks of treatment and then discontinued. Two eszopiclone subjects withdrew from the study due to metallic taste. Both groups showed a numerical decrease in ISI scores; this was not significant for the suvorexant group, although these subjects had a lower ISI at study entry. Neither group reported rebound insomnia, and there were no changes in Pittsburgh Sleep Quality Index (PSQI) scores or increased adverse events compared to baseline. Depression and anxiety scores tended to improve, although this was only significant for reduced anxiety in the eszopiclone group [33].

A number of studies have reported on discontinuing BZDs, either by tapering or by adding on other classes of hypnotics or other medications while tapering the BZD. A meta-analysis including six studies (322 subjects, mean age~64) failed to find an effect of melatonin on benzodiazepine discontinuation, and sleep quality effects were inconsistent [34]. A systematic review included four studies (81 subjects, most >65 years of age) on the use of melatonin and melatonin agonists on BZD or other hypnotic withdrawal rates. In the two placebo-controlled studies, melatonin use resulted in total discontinuation rates of 64.3–77.8% compared to rates of 0–25% with placebo, whereas in the two prospective cohort studies, total discontinuation rates ranged from 30.8–65% [35]. These two reviews [34,35] included two overlapping studies; the later review excluded several studies from the earlier meta-analysis due to subjects being drug dependent or for other methodological concerns [35]. 

A retrospective, observational study of 170 individuals over 50 years of age with major depression and insomnia found that almost half of them were able to switch from a benzodiazepine or Z-drug to controlled-release melatonin [36]. Those who were successful had less anxiety at baseline, had used hypnotics for a shorter period of time, and had started antidepressants earlier [36]. 

Several other pharmacological approaches have been used to discontinue the use of benzodiazepine hypnotics in people with insomnia. A large study had subjects with chronic insomnia (*n* = 1002, mean age~44 years) using hypnotic or anxiolytic benzodiazepines for sleep randomized to one of three treatments: gradual substitution with zopiclone, abrupt substitution of zopiclone, or continuation of current benzodiazepine (unbeknownst to subject) [37]. During the 5-week substitution phase, both zopiclone groups reported improvements in sleep, although they also reported more benzodiazepine withdrawal symptoms than the group that remained on their benzodiazepine; withdrawal symptoms were greater in the abrupt discontinuation group. During the 2-week discontinuation phase that followed, however, withdrawal symptoms were significantly greater, and sleep quality was significantly worse in the group that had remained on a benzodiazepine [37]. Similarly, a randomized, double-blind study of 24 subjects with insomnia and chronic use of the benzodiazepine flunitrazepam found that switching them to zopiclone prior to discontinuation of the hypnotic resulted in better sleep parameters as measured by polysomnography and actigraphy, as well as self-reported measures [38]. There were no significant differences in benzodiazepine withdrawal symptoms between the two groups as measured by self-report questionnaires. Other studies using zopiclone for benzodiazepine discontinuation have reported similar results [24].

In general, both withdrawal and rebound are more severe with shorter-acting agents. Triazolam, in particular, has been shown to lead to increased sleep latency, increased wakefulness after sleep onset (WASO), and decreased sleep efficiency (SE) and total sleep time (TST) upon acute discontinuation, whereas rebound indices for these variables were either not indicated or not significant for temazepam and flurazepam [26]. Rebound insomnia was not observed upon discontinuation of temazepam in insomnia subjects [39]. Quazepam, another longer-acting benzodiazepine, was also reported to cause minimal rebound or withdrawal symptoms [27,40]. Kales et al. compared the effect of acute discontinuation of quazepam or temazepam on rebound insomnia. They found a non-significant increase in sleep latency and wakefulness after sleep onset on the first night after stopping temazepam. With quazepam, sleep latency was still significantly improved in comparison to baseline on the first discontinuation night [41]. 

A sleep laboratory study of insomnia subjects reported significant rebound insomnia upon discontinuation of triazolam, but not quazepam, over a 7-day withdrawal period [42]. Tapering triazolam over several nights, however, decreased rebound insomnia compared to abrupt discontinuation [43]. Another study assessing rebound insomnia of five benzodiazepine drugs in a sleep laboratory over three nights following hypnotic withdrawal (while being given a placebo) found that the short-acting hypnotics triazolam, midazolam, and lormetazepam had higher rates of rebound insomnia over the three nights following withdrawal than the longer-acting drugs flurazepam and quazepam; the latter were not statistically different from a group with placebo-only administration [44]. Furthermore, rebound insomnia was not observed over the fifteen nights following withdrawal of flurazepam and quazepam as determined by polysomnography. In a double-blind study of insomnia subjects assessed over one week following drug withdrawal, rebound insomnia was reported following triazolam but not quazepam discontinuation [45]. A randomized, double-blind study used subjective self-reporting to compare every-other-night dosing effects of triazolam, quazepam, or placebo. The results showed that though both drugs improved sleep on treatment nights, rebound insomnia was reported on off nights in the triazolam group [46]. Temazepam, which has an intermediate half-life, is also associated with rebound insomnia for up to several nights following discontinuation [47]. A sleep laboratory study of healthy subjects treated with medication for 4 weeks found greater rebound insomnia on the two nights following triazolam withdrawal than following withdrawal of either zolpidem or zopiclone [48], suggesting that not only the half-life but the type of BzRA may impact rebound insomnia.

Even short-term use of BZDs can lead to rebound insomnia upon acute withdrawal. In a polysomnography study comparing the effects of acutely withdrawing insomnia subjects twice from hypnotics after only a few days of treatment with an intermittent dosing protocol [49], significant rebound insomnia was consistently seen following triazolam withdrawal, but only after the second temazepam withdrawal, concluding that rebound insomnia may be seen with both drugs. Though short-acting hypnotics can lead to rebound insomnia immediately following withdrawal, longer-acting BZDs may sometimes result in rebound insomnia following several days of discontinuation. For example, in a sleep lab study of six insomnia subjects, withdrawal of clonazepam led to significant rebound insomnia on the third night following withdrawal, as assessed by increased waking after sleep onset and increased total wake time scores [49]. 

#### 3.1.3. Best Practices for BZD Discontinuation 

Clinical data suggest that BZDs should be tapered, preferably with some kind of behavioral therapy (e.g., CBT-I) or other support in place (Table 2). No data were found to determine whether switching to a longer half-life hypnotic drug decreases withdrawal or rebound insomnia symptoms. Currently, there is no consensus in the literature as to what the tapering schedule should be, although most studies reported reducing the dose by ~10–25% increments at intervals of one to several weeks.

An evidence-based clinical practice guideline (systematic review; used GRADE approach) from the Family Physicians of Canada on deprescribing BzRAs for insomnia recommended that deprescribing (slow tapering) should be offered to all adults > 65 years old regardless of duration of use and suggested to adults 18–64 who have used benzodiazepine receptor agonists > 4 weeks. The recommendations are applicable to individuals with primary insomnia and to those with comorbid insomnia whose underlying comorbidities are effectively managed [50].

Another systematic review of interventions to deprescribe BZDs and other hypnotics in older adults (>65 years; seven studies) reported that pharmacological substitution or tapering with psychological support was more effective than patient education with tapering, and that deprescribing did not lead to an increase in withdrawal symptoms or a decrease in sleep quality [51]. A systematic review of BZD discontinuation in older adults with insomnia, anxiety, or depression found that all modalities of discontinuation (taper alone, taper plus cognitive behavioral therapy, taper plus medication substitution) were effective, and results were independent of dose or duration of hypnotic use [52]. 

A systematic review and meta-analysis of psychosocial interventions for BZD hypnotic discontinuation (eight studies in the meta-analysis) found that short-term (<3 mos) CBT-I plus gradual tapering was more effective than gradual tapering alone for discontinuation, but there was no difference in long-term efficacy of CBT-I at 12 months [53].

### 3.2. Z-Drug Discontinuation 

The non-benzodiazepine GABA-A receptor positive allosteric modulators, commonly called “Z-drugs”, include the sedative hypnotics zolpidem, zaleplon, zopiclone, and eszopiclone. Zopiclone is not commercially available in the United States. Thus, this section summarizes the literature concerning discontinuation of zolpidem (both immediate release and extended release), zaleplon, and eszopiclone (Table 1).

#### 3.2.1. Risk of Adverse Effects of Z-Drug Discontinuation

##### Zolpidem 

Multiple investigators have examined the effects of deprescribing nightly dosing of zolpidem at the standard doses of 10 mg immediate release or 6.25 mg extended release either as a monotherapy or as a later add-on treatment to ongoing selective serotonin reuptake inhibitors (SSRI). Studies show that abrupt discontinuation of standard doses of zolpidem are followed by a deterioration in sleep on the first night; some studies found that sleep was worse than baseline (i.e., “rebound insomnia”) on night 1 [48,54,55,56], whereas others report worsening of sleep on night 1, but without meeting criteria for rebound insomnia [57,58]. Sleep metrics in these studies re-stabilized towards pre-treatment baseline by night 2. Though most studies did not systematically assay for benzodiazepine-type withdrawal symptoms after discontinuation of standard zolpidem doses, significant increases in daytime withdrawal symptoms following assessment with the Tyrer Benzodiazepine Withdrawal Questionnaire have been reported [57].

One study examined tapering of either immediate-release or extended-release zolpidem followed by immediately transitioning to the DORA lemborexant 5 or 10 mg. Of 53 subjects enrolled in the study, 81% successfully transitioned to lemborexant. The primary adverse events reported were somnolence or abnormal dreams [59].

##### Zaleplon 

Two RCTs have contrasted the self-reported discontinuation effects of zaleplon 5, 10, or 20 mg versus zolpidem 5 or 10 mg in a total of 1135 participants across both trials [60,61]. Both doses of zolpidem were associated with higher rates of rebound insomnia for sleep latency and TST on night 1, whereas zaleplon discontinuation was free of rebound insomnia [60]. Zaleplon 10 mg was not associated with rebound insomnia for sleep latency or TST, while zolpidem met the criteria for rebound insomnia for these parameters only on night 1 [61]. Furthermore, daytime symptoms of benzodiazepine-type withdrawal were seen in 7.1% of persons discontinued from zolpidem but were not seen in the zaleplon group. Investigation of rebound insomnia in 170 persons > 65 years old upon discontinuation of open-label zaleplon 5–10 mg, revealed sleep latency, TST, and number of awakenings all showed a statistical worsening on night 1 but did not meet the criteria for rebound insomnia [62]. A 2009 review paper concluded that “the abrupt discontinuation [of zaleplon] does not appear to produce rebound insomnia or withdrawal symptoms in adult or elderly insomnia patients” [56].

##### Eszopiclone 

Three RCTs of eszopiclone 2 or 3 mg examined the effects of single-blind runout. Polysomnographic study data showed an approximately 50% loss of benefit from the 3 mg dose compared to placebo for sleep latency and sleep efficiency on night 1 of placebo substitution, and the 2 mg dose showed rebound insomnia for sleep efficiency and wake after sleep onset on night 1 of placebo. No dose showed evidence of rebound by night 2 of placebo substitution [63]. A similarly designed study of eszopiclone 2 mg in adults >65 years old demonstrated partial loss of sleep benefit by self-report on night 1 of placebo substitution but without meeting the criteria for rebound insomnia. Reported sleep had stabilized by the end of the 7 days of observation during placebo runout [64]. Assessment for evidence of symptoms of benzodiazepine-type daytime withdrawal in 123 adults receiving open-label eszopiclone with abrupt discontinuation revealed 10.5% of the sample had “clinically relevant” symptoms of withdrawal [65]. 

The pattern of discontinuation effects just described is observed when eszopiclone is given as a monotherapy for people with insomnia in the absence of psychiatric disorders. In contrast, two RCTs of eszopiclone in persons with depression or generalized anxiety disorder (GAD) have found that when eszopiclone (versus placebo) is initiated simultaneously with an SSRI, there is little or no loss of benefit when the eszopiclone is abruptly discontinued [66,67]. In fact, subjects who were assigned to eszopiclone during the blinded randomized phase self-reported superior sleep during the placebo runout compared to those who were assigned to placebo during the randomized phase. By implication, treating depression or anxiety-related insomnia with an SSRI may assist in the resolution of insomnia after 8 weeks of treatment, allowing for the successful discontinuation of the hypnotic with preservation of the insomnia benefit.

The findings from individual studies of eszopiclone were further supported by a 2018 meta-analysis of 14 eszopiclone RCTs finding no evidence of rebound insomnia for sleep latency or wake after sleep onset [68]. The reports from these single studies are consistent with the conclusion that “clinical trials of eszopiclone have found little evidence of the development of…. withdrawal effects” [56].

#### 3.2.2. Best Practices for Z-Drug Discontinuation

Most of the RCTs examining the acute sleep and daytime effects of stopping Z-drugs have not tapered the Z-drug and instead have used abrupt placebo substitution. Tapering is always medically necessary when the individual is taking excessively high doses of Z-drugs, in order to avoid severe withdrawal reactions, which might include seizures (Table 2).

A tapering schedule for hypnotics of a 25% reduction in the dose per week has been described [69]. For the most part, little is known about participants’ attitudes towards tolerating deprescribing Z-drugs or switching from Z-drugs to a different agent [59]. Zolpidem, and to a lesser extent eszopiclone, seem to be associated with at least one night of rebound insomnia, and hence, tapering these medications might be expected to improve tolerance for switching agents. Older individuals might be more willing to stop their present hypnotic if they are advised of the risk of continuing with their present regimen [70]. In general, people want to be informed of alternative strategies for managing their insomnia if they are being advised to stop their present hypnotic [71], and the deprescribing plan may be more successful if the individual is fully educated about what to expect during discontinuation [51,71,72]. The introduction of CBT-I may also be helpful in transitioning people off of hypnotic medication [23,73].

### 3.3. DORAs, Doxepin, and Ramelteon Discontinuation

This section contains a summary of the literature regarding the degree to which loss of benefits, rebound insomnia, and withdrawal symptoms occur in people with insomnia following abrupt discontinuation of the dual orexin receptor antagonists (DORAs; suvorexant, lemborexant, daridorexant), the melatonin receptor agonist ramelteon, and low doses of the tricyclic antidepressant doxepin (Table 1).

#### 3.3.1. Risk of Adverse Effects of DORAs, Doxepin, or Ramelteon Discontinuation

##### DORAs

We reviewed a clinical trial of 521 subjects aged 18 years or older with primary insomnia by Diagnostic and Statistical Manual (DSM)-IV-TR criteria who were randomized to receive nightly suvorexant (40 mg for individuals younger than 65 years, 30 mg for those aged 65 years or older) or placebo at a 2:1 ratio for 1 year with a subsequent 2-month randomized discontinuation phase in which subjects on suvorexant either continued suvorexant or were abruptly switched to a placebo [74]. Additionally, we found two randomized, double-blind, placebo-controlled, parallel-group, 3-month trials in nonelderly (18–64 years) and elderly (≥65 years) subjects with insomnia who were randomized to suvorexant doses of 40/30 mg (nonelderly/elderly) and 20/15 mg (nonelderly/elderly) [75,76]. Analyses of rebound and withdrawal symptoms were assessed during three nights of post-double-blind treatment runout. Withdrawal symptoms were assessed with the Tyrer Withdrawal Symptom Questionnaire for all studies of suvorexant considered. It is important to note that most of the dosage studies are above those approved by the U.S. FDA for the treatment of insomnia. 

There was no evidence of withdrawal effects for any studies except for one of the two suvorexant 3-month studies at one dosage [75]. In one of these trials, there were no significant differences in the numbers of subjects meeting the pre-specified withdrawal criteria on any night or across the three nights for the comparisons of the suvorexant → suvorexant groups versus the suvorexant → placebo groups. The pattern of findings was generally similar in trial 2, except significantly more individuals in the suvorexant 40/30 mg → placebo group met the pre-specified withdrawal criteria on the first night compared with the suvorexant 40/30 mg → suvorexant 40/30 mg group, but not on the other nights or across the three nights. In both trials, no adverse events that might potentially be associated with withdrawal based on a predefined list (e.g., irritability, palpitations) were reported. 

Rebound insomnia was assessed by different methods across studies. In general, no studies identified rebound insomnia in terms of mean values on sleep outcomes. However, rebound insomnia was detected in suvorexant studies assessing the percentage of individuals experiencing worsening in sleep parameters with discontinuation to placebo vs. continued drug, which is presumably a more sensitive measure, though the percentage of individuals experiencing worsening was not assessed for some of the other treatments. In the same report in which withdrawal symptoms were noted for one of two studies of suvorexant, rebound insomnia was noted [75,76]. Analyses of rebound insomnia during the first three nights of the runout for trial 1 indicated that there were no statistically significant differences for self-reported TST or time to sleep onset on any one night or on any of the three nights for the comparisons of the suvorexant → placebo groups with the placebo → placebo group. However, the proportions of subjects meeting the rebound insomnia criterion on most comparisons, although low in all groups, were numerically greater in the suvorexant → placebo groups compared with the placebo → placebo group. There was a similar pattern of findings in trial 2 in which the proportions of patients meeting the rebound insomnia criterion on self-reported TST were significantly greater in the suvorexant 40/30 mg → placebo group compared with the placebo → placebo group.

For suvorexant at dosages higher than those FDA approved for insomnia, sleep worsened compared to the end of double-blind treatment for 2 months in terms of mean self-reported TSO and TST, but the means remained above baseline throughout this period, though no statistical comparisons were made to baseline or the end of double-blind treatment [74].

One study of lemborexant was available for consideration [77]. This was a 12-month, randomized, double-blind, parallel-group, phase 3 study divided into two treatment periods. In treatment period 1 (first 6 months), subjects (*n* = 949, full analysis set) were randomized to daily placebo, lemborexant 5 mg (LEM5), or lemborexant 10 mg (LEM10). In treatment period 2 (second 6 months), placebo subjects were re-randomized to LEM5 or LEM10, and subjects initially randomized to lemborexant continued their assigned treatment (LEM5, *n* = 251; LEM10, *n* = 226). Sleep was evaluated for a 2-week period following discontinuation at the end of the 1-year treatment period [77,78]. Those on lemborexant medication for 1 year stayed statistically significantly better than baseline for 2 weeks post-discontinuation and were significantly worse than at the end of double-blind treatment for two weeks after discontinuation for self-reported sleep onset latency (SOL), but for self-reported WASO, subjects were only significantly worse than at the end of double-blind treatment for the first week [77,78]. During the second week, the mean of WASO was higher but not above 95% confidence limits.

A single study of daridorexant discontinuation was also identified [79]. The study included adults with insomnia disorder who completed a 12-week double-blind placebo-controlled trial in which they were randomized to daridorexant (10 mg/25 mg/50 mg) or placebo. Those randomized to daridorexant remained on their respective treatment for an additional 40 weeks, whereas those randomized to placebo were re-randomized to daridorexant 25 mg or placebo. The 52-week treatment period was followed by a 7-day placebo runout in which rebound and withdrawal were assessed. Daridorexant was found to be associated with sleep that was statistically significantly better than baseline during 1 week of placebo discontinuation, and though sleep during this period was worse than at the end of double-blind treatment, this was not assessed in terms of statistical significance [79].

##### Low-Dose Doxepin

There was one study of doxepin available for analysis [80]. This was a randomized, double-blind, parallel-group, placebo-controlled trial in subjects meeting DSM-IV-TR criteria for primary insomnia who were randomized to 35 days of nightly treatment with doxepin 3 mg (*n* = 75), doxepin 6 mg (*n* = 73), or placebo (PBO; *n* = 73), followed by two nights of single-blind PBO to evaluate discontinuation effects. Withdrawal was assessed with the Benzodiazepine Withdrawal Symptom Questionnaire. Doxepin was associated with statistically improved sleep during a 2-day discontinuation period compared with baseline, and though mean sleep values were worse during discontinuation than during the end of double-blind treatment, this was not tested statistically [80].

##### Ramelteon

Lastly, four studies of ramelteon were identified and considered. The first of these consisted of a 6-month, randomized, double-blind, placebo-controlled study of 451 adults (age ≥ 18 years) with chronic primary insomnia who were randomized to ramelteon 8 mg or placebo [81]. Sleep was evaluated by polysomnography and morning questionnaires, including one on nights 1 and 2 of the placebo runout, during which rebound insomnia and withdrawal effects were evaluated. The study found no next-morning residual effects, rebound insomnia, or withdrawal symptoms upon discontinuation of ramelteon. The second study consisted of a single-blind, flexible-titration, multicenter study incorporating placebo run-in and runout periods in which 190 adults with chronic insomnia received ramelteon 4 or 8 mg, titrated up to 16 mg, if necessary, for 24 weeks [82]. There was no evidence of rebound insomnia. The third study was a randomized, multicenter, double-blind, placebo-controlled trial of 5 weeks of nightly ramelteon treatment (8 mg or 16 mg) in adults (*n* = 405) with primary chronic insomnia in which rebound and withdrawal were studied during a two-day placebo runout period [83]. Again, there was no evidence of rebound insomnia or withdrawal. The fourth study was a randomized, double-blind, placebo-controlled 35-night outpatient trial including older adults (>or =65 years; *n* = 829) with chronic insomnia randomized to placebo, ramelteon 4 mg, or ramelteon 8 mg nightly for 5 weeks followed by a 1-week placebo discontinuation phase [84]. Withdrawal was assessed with the Benzodiazepine Withdrawal Symptom Questionnaire for all ramelteon studies. In the ramelteon studies by Roth and Mayer, latency to persistent sleep remained statistically significantly better than baseline for 1 week of discontinuation and was not statistically significantly different than the end of double-blind treatment [81,84]. There was no evidence for rebound insomnia or withdrawal.

#### 3.3.2. Best Practices for DORAs, Doxepin, or Ramelteon Discontinuation 

By and large, the treatments studied were associated with worsening of sleep during placebo discontinuation from the end of double-blind treatment, but sleep remained better than baseline during discontinuation. The lengths of periods studied and whether statistical comparisons were carried out varied among the studies. Taken together, there is no signal indicating a safety concern for any of the medications studied in this section, nor any evidence for substantial or consistent rebound. Even in the suvorexant studies in which there was evidence for an increase in the percentage of individuals with worsening of sleep on discontinuation versus those continuing medication, the mean values of all key insomnia outcomes were improved over baseline. As a result, it appears that the data do not support the need for a taper or the institution of any other measures to ensure safety in the discontinuation of any of these medications (Table 2). Further, there seems to be no need for such measures when trying to switch from these agents, other than setting expectations about the possibility that in some individuals, sleep may transiently worsen.

### 3.4. “Off-LABEL” Drugs in the Treatment of Insomnia

This section includes a summary of the available data from the literature about deprescribing and discontinuing medications that are not FDA-approved for insomnia disorder but are prescribed in clinical practice to treat insomnia.

#### 3.4.1. Risk of Adverse Effects of “Off-Label” Drug Discontinuation

Medications from various pharmacodynamic classes are often prescribed specifically for the treatment of insomnia disorder despite their having neither an FDA approval for this indication nor supportive clinical trial evidence regarding their efficacy and safety in this population. Accordingly, such medication use may be described as “off-label”. There are only a few controlled studies for the sleep-promoting effects of these medications, predominantly concerning antidepressants and antipsychotics; even fewer studies have assessed discontinuation methods and effects, either for sleep parameters upon abruptly stopping a medication or newly emergent withdrawal symptoms (Table 1).

##### Trazodone

The Desyrel (trazodone hydrochloride) Prescribing Information for the medication’s use as an antidepressant states, “adverse reactions may occur upon discontinuation of Desyrel”, and the associated Medication Guide advises patients that “symptoms of withdrawal can include anxiety, agitation, and sleep problems” [85]. Placebo runout periods have been incorporated into protocols in studies of the controlled-release form of trazodone for its FDA-indicated use. Six individuals with chronic insomnia were studied with a single-blind protocol including a 7-day placebo phase, a 6-week period of increasing doses of trazodone controlled-release up to 150 mg nightly, and a 7-day single-blind placebo period [86]. Polysomnographic studies were performed on five occasions (placebo baseline, three during the active treatment period, and after drug discontinuation). The active treatment was associated with a significant increase in slow-wave sleep and a decrease in NREM stage 2, as well as decreases in cyclic alternating pattern time and rate. These changes returned to baseline following discontinuation. A visual analog scale indicated increased sleep quality during the active treatment period with a return to the premedication level upon drug discontinuation. Actigraphy and insomnia rating scores (Athens Insomnia Scale) were assessed in people with primary insomnia, with (*n* = 14) and without (*n* = 15) increased depression scores treated with controlled-release trazodone (doses 25 to 150 mg) for 3 months and during a 1-month runout phase with no sleep-promoting medication. Only subjects with increased depression scores demonstrated significant deterioration of sleep according to the insomnia rating scale [87]. 

Published case reports describe a variety of potential discontinuation symptoms associated with trazodone. A case study described a 38-year-old man treated with trazodone up to 300 mg daily that was abruptly discontinued following two episodes of priapism [88]. Within 36 h, he reported restless sleep, nightmares, “near panic”-level anxiety, and periods of depersonalization, all of which resolved within 5 days [89]. A 42-year-old man was taking trazodone 600 mg that was discontinued over a period of 5 days due to lack of improvement in his mood. Within 24 h of discontinuation, he developed nausea, recurrent vomiting, and diaphoresis. A 65-year-old woman was prescribed trazodone 250 mg nightly that subsequently was discontinued over 3 days. She developed nausea and vomiting within 15 h after her last dose. Three case studies were described of individuals treated with trazodone (two taking up to 300 mg and one taking up to 150 mg) with gradual dose reductions over several weeks who developed symptoms including insomnia, nausea and vomiting, diarrhea, lassitude, myalgia, and restless legs [90]. The authors suggested the effects of trazodone and its metabolite meta-chlorophenylpiperazine (m-CPP) on the serotonin system resulted in noradrenergic rebound after discontinuation and recommended that trazodone should be tapered off “at a very slow rate”.

##### Mirtazapine and Esmirtazapine

Mirtazapine has not been evaluated for discontinuation effects in individuals with insomnia; however, potential withdrawal symptoms are noted in the FDA-approved Prescribing Information, which states, “There have been reports of adverse reactions upon the discontinuation of REMERON/REMERONSolTab (particularly when abrupt), including but not limited to the following: dizziness, abnormal dreams, sensory disturbances (including paresthesia and electric shock sensations), agitation, anxiety, fatigue, confusion, headache, tremor, nausea, vomiting, and sweating, or other symptoms which may be of clinical significance.” The label goes on to recommend a gradual reduction in the dose [91].

Though mirtazapine has not been assessed for efficacy, safety, or discontinuation effects in the treatment of insomnia, several studies have been performed with the investigational compound esmirtazapine, the S-enantiomer of mirtazapine that has similar pharmacologic properties. One group performed a randomized placebo-controlled trial of esmirtazapine doses vs. placebo in a 2-week active treatment period followed by a single-blind 1-week placebo runout [92]. The protocol was completed by 463 primary insomnia participants. During the placebo runout, the sleep parameters (TST, SOL, and WASO) demonstrated improvement relative to baseline on most nights, with no evidence of rebound insomnia or withdrawal symptoms. 

Similarly, 366 primary insomnia participants took part in a randomized placebo-controlled trial of esmirtazapine doses vs. placebo in a 6-week active treatment period followed by a 1-week single-blind placebo runout [93]. During the placebo runout, the polysomnography (PSG) parameters (TST, SOL, and WASO) and subject-reported assessments demonstrated significant improvement relative to baseline and no evidence of rebound insomnia or withdrawal symptoms. 

Another randomized placebo-controlled trial comprised of 457 adults with chronic insomnia studied the effects of esmirtazapine 4.5 mg or placebo for 6 months followed by a 7-day discontinuation period [94]. Following the treatment period, the esmirtazapine group was double-blind re-randomized 1:2 to continue esmirtazapine (*n* = 65) or abruptly switch to placebo (*n* = 137). Discontinuation effects were assessed with a daily diary, withdrawal symptom questionnaire, and adverse event monitoring. The authors reported no evidence of rebound insomnia (based on TST, SOL, and WASO) or withdrawal symptoms upon treatment discontinuation. It was noted that the efficacy of esmirtazapine during the treatment period appeared to be sustained during the discontinuation phase, as the improvement from pre-treatment was higher for the esmirtazapine group.

##### Doxepin (Antidepressant-Approved Doses) 

Among tricyclic antidepressants (TCAs), discontinuation effects have been assessed in clinical trials with doxepin (doses greater than FDA-approved 3 and 6 mg) and trimipramine. The efficacy and safety of doxepin 25 to 50 mg was compared with placebo in a randomized trial that included a 4-week active treatment period followed by a 2-week single-blind placebo runout [95]. The participants met the criteria for primary insomnia, with doxepin and placebo groups each including 20 completing participants. PSG studies were performed on selected nights, including the first three nights and the final night of the runout period. None of the assessed sleep parameters (TST, WASO, SE, or sleep stage 2 percentage) demonstrated rebound effects in the doxepin group, although individual participants with severe rebound insomnia were significantly more frequent compared to placebo. Mean SE and WASO values at the end of the 2-week placebo runout were significantly better than at baseline. 

##### Trimipramine

Trimipramine was assessed in a single-blind pilot study (mean dose 166 mg after a stepwise increase) in individuals with primary insomnia (15 completers) during a 28-day active treatment period followed by a 2-week placebo runout [96]. Self-reported and objective assessments included questionnaires and home EEG recordings, including at 4 and 14 days following drug discontinuation. Self-reported indicators did not show rebound, and there was no increase in withdrawal symptoms during discontinuation. Another study compared the effects of trimipramine (mean dose 104 mg) to placebo and lormetazepam in a randomized double-blind controlled study of 55 individuals with primary insomnia with an active treatment period of 4 weeks followed by a 2-week single-blind placebo runout [97]. PSG measurements were taken on day 2 and day 12 following the medication discontinuation. Relative to baseline, the participants in the three groups did not demonstrate withdrawal effects, though the trimipramine participants did have higher scores on the Withdrawal Symptom Scale during discontinuation. During discontinuation, PSG testing showed a significant decrease in TST among the trimipramine and placebo participants but not worse than baseline. Self-reported ratings of sleep quality decreased in all three groups but stayed above baseline. 

##### Quetiapine

Though quetiapine discontinuation effects are not specifically addressed in insomnia populations, they have been described in broader patient populations. A meta-analysis found “an association between rapid cessation of quetiapine and onset of somatic symptoms such as nausea, vomiting, agitation, restlessness, diaphoresis, irritability, anxiety, dysphoria, sleep disturbance, insomnia, tachycardia, hypertension and dizziness” [98]. Case studies on quetiapine discontinuation, though considered poor quality, report insomnia or sleep disturbance following discontinuation in 27% (*n* = 4) of the cases. One 21-year-old woman who abruptly stopped quetiapine 300 mg became diaphoretic within one day, and within two days, she experienced light-headedness, nervousness, and nausea and vomiting [99]. Her symptoms resolved after a return to the 300 mg dose followed by a slow cross-taper with an alternate antipsychotic medication. A 36-year-old woman was described with “incapacitating quetiapine withdrawal,” after reducing quetiapine 100 mg at bedtime to 50 mg [100]. Within one day, she developed nausea, dizziness, headache, and anxiety, with her symptoms resolving over a period of several days with the use of antiemetics [100]. Quetiapine Prescribing Information states that, “acute withdrawal symptoms, such as insomnia, nausea, and vomiting have been described after abrupt cessation of atypical antipsychotic drugs, including quetiapine” [101]. Discontinuation dyskinesia can also be observed when stopping quetiapine. 

#### 3.4.2. Best Practices for Discontinuation of Off-Label Drugs for Insomnia

A great deal of information is available concerning the rapid discontinuation of antidepressant medications and withdrawal effects. In 1984, Dilsaver and Greden described antidepressant withdrawal syndrome, noting that symptoms may include “sleep disturbance characterized by excessive and vivid dreaming and initial and middle insomnia”. [102]. More recently, a review on antidepressant discontinuation syndrome noted that key symptoms that may last days to months include insomnia, flu-like symptoms, mood disturbances, and paresthesia [103]. The authors also state that discontinuation of TCAs and atypical antidepressants can result in sleep disturbances, nightmares, and vivid dreams, and they note an intermediate risk of antidepressant discontinuation syndrome with amitriptyline and trazodone and a low risk for mirtazapine. Indeed, discontinuation effects in serotonin and norepinephrine reuptake inhibitor (SNRI) treatment can be seen for weeks and months in some patients. A recommendation to gradually reduce the dose when discontinuing these off-label medications is consistent with each drug’s Prescribing Information (Table 2). However, whether there is a lower threshold below which tapering is unnecessary remains an open question.

## 4. Discussion

Medications to treat insomnia are some of the most commonly prescribed treatments in the world [16]. Because the causes and comorbidities of insomnia are inherently complex, and the disease itself can wax and wane in presence or severity, medication switching or discontinuation is common. Concerns regarding potentially related rebound insomnia, withdrawal symptoms, or psychological dependence can make these transitions challenging for both the prescribing practitioners and their patients. We sought to provide clarity to best practices when making these insomnia medication transitions to maximize successful treatment outcomes and minimize problematic issues. To our knowledge, this is the first evidence-based clinical guideline to address the issue of deprescribing or switching medications in individuals with insomnia. 

In general, BZDs should be tapered when discontinuing or switching to a different insomnia medication. Reducing the medication by 10–25% over weeks to months ensures the smoothest and safest possible transition. BZDs with a short half-life and/or higher doses of these medications warrant a more conservative approach, whereas those with longer half-lives and/or lower doses allow for a more rapid transition. Discontinuing “Z-drugs” in general is not problematic beyond 1-2 days, regardless of how discontinuation is implemented. However, when supratherapeutic doses have been taken, a 25% reduction per week over 4 weeks is recommended. Ramelteon, insomnia-related doses of doxepin, and DORAs require no special considerations upon discontinuation or medication switching. There was a general lack of meaningful evidence examining the discontinuation of antidepressant and antipsychotic medications used “off-label” for insomnia specifically in individuals with insomnia. Therefore, we defer to the specific medication’s Prescribing Information when discontinuing these drugs. 

A number of issues created challenges to our recommendation process. First was settling on the operational definition of “rebound insomnia”. Insomnia is not a static disease. In some cases, it may remit upon discontinuation of therapy, but symptoms may also worsen. CBT-I has demonstrated good durability of beneficial effects following discontinuation, but sustained improvement is less predictable with insomnia pharmacotherapy [104]. As such, we expect in many cases baseline insomnia symptoms will return following discontinuation of an insomnia therapy. Even if the duration criterion for insomnia is met (i.e., at least 3 months of symptoms), any particular case of insomnia may be precipitated by an identifiable stressor that, when removed, will allow the insomnia to regress without any further treatment of any kind. There is also the circumstance of insomnia in the setting of an episode of depression or GAD; and in this paper, we mention clinical trials showing that when SSRIs and hypnotics are given together, the hypnotic might be removed after 8 weeks without regression of the insomnia improvement. Considering these factors, we deem “rebound insomnia” to mean worsening of insomnia symptoms compared to baseline upon medication discontinuation. For FDA-approved insomnia therapies, short and mid-acting BZDs (e.g., triazolam, temazepam) and the non-benzodiazepine GABA-A receptor modulators zolpidem (and to a lesser extent eszopiclone) had evidence showing rebound insomnia [24,56,105]. For zolpidem and eszopiclone, this was only observed for one night, whereas rebound insomnia may persist for longer upon discontinuation of the aforementioned benzodiazepines. Off-label use of the antidepressant trazodone and the antipsychotic quetiapine for insomnia also produced rebound insomnia upon discontinuation [89,98]. None of the dual orexin receptor antagonists (DORAs) consistently produced rebound insomnia after stopping the medication. 

Other challenging issues included the impact of the insomnia medication dose, frequency of administration, duration of use, or taking multiple insomnia medications on subsequent potential rebound insomnia or withdrawal symptoms upon discontinuation. Regarding dose, although our evidence base included a few case reports of individuals taking many times the maximum FDA-approved dose of a medication (e.g., zolpidem, trazodone), the evidence primarily involved individuals taking FDA-approved doses [89,106]. One caveat is BZDs, where higher doses may predispose to more withdrawal symptoms upon discontinuation. We found no evidence specifically addressing the impact of frequency or duration of medication administration on discontinuation or switching. We also found little evidence of how the administration of multiple insomnia medications concurrently influences stopping or switching insomnia medications. Therefore, our switching or deprescribing recommendations are most applicable to nightly use of FDA-approved doses of single insomnia medications taken for any duration of time. Because untoward effects of medication switching or deprescribing are likely to be worse following nightly dosing than intermittent dosing, our recommendations are based on a conservative definition of insomnia medication use and are therefore applicable to situations of intermittent use as well.

We found a paucity of evidence addressing switching between specific insomnia medications or from one class of medications to another. Clearly, future insomnia medication clinical trials should be extended to assess the impact of transitions from a current therapy to the medication under investigation. Regardless, one study showed switching from a benzodiazepine to lemborexant went smoothly, regardless of whether the medications were changed abruptly, cross-titrated, or lemborexant was added prior to titrating off the benzodiazepine [32]. Other research shows adding an insomnia medication prior to discontinuing a benzodiazepine risks oversedation [31]. The impact of rebound insomnia or withdrawal symptoms upon insomnia medication switching was of particular concern regarding impacts on perceptions of subsequent insomnia medication efficacy. Individuals often judge an insomnia medication’s performance based on their initial experience, but rebound insomnia or lingering withdrawal symptoms from the previous insomnia medication may incorrectly be ascribed to the new medication. This can result in premature discontinuation of the new medication and a potential missed opportunity to improve insomnia symptoms. For this reason, practitioners should explain this phenomenon to individuals up front when switching insomnia medications to ensure an adequate treatment trial of the ensuing medication. 

Off-label prescribing with the intention to promote sleep, whether for difficulty with sleep onset or maintenance, has historically been done with sedating antidepressants and more recently with atypical antipsychotics. In past decades, amitriptyline and doxepin (in doses above the currently approved 3 and 6 mg formulations) were common choices, replaced during more recent decades by trazodone and mirtazapine. Currently, trazodone is the medication most frequently prescribed for insomnia, ranking far above the FDA-approved insomnia medications at number 21 in the latest (2020) list of drugs prescribed for all conditions. Among antipsychotics, quetiapine is the predominant selection. When any of these medications have been prescribed to treat insomnia symptoms, clinical decision making about discontinuation should take into consideration whether deterioration in sleep or new-onset withdrawal symptoms might be anticipated. Though a paucity of evidence exists regarding deprescribing or switching from these medications to other treatments in individuals with insomnia, abundant literature endorses gradual dose reductions rather than abrupt discontinuation, in order to mitigate withdrawal effects of these medications in individuals with non-insomnia-related indications for treatment [102,103]. 

Because antidepressant and antipsychotic medications have different mechanisms of action, cross-tapering (e.g., tapering off one medication while coincidentally ramping up the dose of the other) should be possible as long as problematic drug interactions are not an issue. Doses are typically lower when using antidepressants or antipsychotics to treat insomnia than other illnesses. Yet, researchers have not studied the impact of antidepressant and antipsychotic dose on rebound insomnia and withdrawal symptoms upon discontinuation. With this in mind, individualized dose reduction recommendations should be implemented when stopping antidepressants and antipsychotics used to treat insomnia. Discontinuation strategies should be individualized and may vary depending on medication dose, pharmacodynamics, pharmacokinetics, and whether another sleep-promoting medication is being cross-tapered. Because literature does not exist regarding deprescribing or switching insomnia therapies in specific insomnia populations with certain comorbidities or complicating factors (e.g., substance abuse), we cannot make tailored recommendations for patients with these specific complicating factors. 

## 5. Final Recommendations

In summary, with the exception of short- and mid-acting BZDs and zolpidem and eszopiclone, particularly at higher doses, we found little overall evidence that deprescribing or switching FDA-approved insomnia medications at standard doses is problematic. Abrupt discontinuation of BZDs at lower doses can cause sleep disturbance and anxiety, among other symptoms, and at higher doses, this can cause seizures or psychosis [24,105]. Higher doses and shorter half-lives portend more difficulties when discontinuing BZDs, and in these circumstances, a taper is recommended (e.g., reducing the dose by 10–25% every week or fortnight until discontinued). Although the duration of treatment is less certain regarding impacts on medication discontinuation, it is reasonable to further proceed cautiously when discontinuing BZDs taken for longer periods of time. For zolpidem and eszopiclone, rebound insomnia may occur, but this is typically the case for only one night and when taken at higher “supratherapeutic” doses [56]. Off-label use of antidepressants and antipsychotics tend to produce withdrawal effects generally, and possible rebound insomnia in some instances (e.g., trazodone, quetiapine) [107,108]. We recommend referring to the Prescribing Information for further guidance when discontinuing these medications. 

Interestingly, for some medications (e.g., daridorexant), it appears that aspects of the therapeutic effect may persist beyond medication discontinuation [64,66,109]. Perhaps this medication fosters greater confidence in one’s “sleep ability” that continues following medication discontinuation. The duration of this effect, if present, is unknown. 

In conclusion, offering a rationale for deprescribing or switching, involving patients in the decision-making process to foster a sense of self-efficacy, and offering alternative therapies with a clear explanation of how they will help accomplish treatment goals can facilitate the medication change and enhance the opportunity for treatment success [51,70,71,72,110]. We conclude that FDA-approved insomnia therapies at standard doses can be deprescribed or switched safely and effectively. In many cases, with the exception of BZDs, zolpidem, eszopiclone, and the off-label use of antidepressants and antipsychotics, medication tapering or “cross-tapering” when switching is not necessary. Among classes of medications, DORAs, selective H1 antihistamines (low-dose doxepin), and melatonin receptor agonists (ramelteon) appear to have the lowest risk of rebound insomnia or withdrawal symptoms upon discontinuation or switching when compared to other classes of FDA-approved medications. Table 2 summarizes our recommendation by insomnia medication class.

## Figures and Tables

**Table 1:** Summarized observed effects of insomnia medication discontinuation or switching.

Initial Drug/Group	No Change in Sleep on Discontinuation	Revert to Baseline or Improved over Baseline	Sleep Worse than Baseline Indicating Rebound	New Symptoms, e.g., Anxiety/ Seizures Indicating Withdrawal
Short-acting BZDs (e.g., triazolam)	Not on night 1	May take several days	Yes	Anxiety, terminal insomnia may occur with use; more severe withdrawal/seizures may occur with abrupt withdrawal, particularly with higher doses
Mid-acting BZDs (e.g., temazepam)	More sleep disruption in some studies	Yes	Sometimes	Not usually at therapeutic doses; withdrawal/seizures may occur with higher doses, abrupt withdrawal
Long-acting BZDs (e.g., quazepam, flurazepam)	Yes	Yes	No	Not usually at therapeutic doses; withdrawal/seizures may occur with higher doses, abrupt withdrawal
Zolpidem	Not on night 1	By the second night	For 1 night	Yes: traditional withdrawal symptoms observed only when using structured instruments. Seizures only with doses exceeding recommended amount.
Zaleplon	Mostly	Yes on night 1	No	No
Eszopiclone	Not on night 1	Yes on night 1	Not generally	Yes when using structured instruments. No when getting spontaneous reports.
DORA—Suvorexant	For higher dosages than FDA approved, means worse than end of double-blind treatment but not tested statistically	For dosages higher than FDA approved, means remained better than baseline for 2 months after discontinuation but not tested statistically	2/3 studies at doses above those used clinically	2/3 studies at doses above those used clinically
DORA—Lemborexant	Significantly worse than end of double-blind treatment for two weeks after discontinuation for SOL, but for WASO, was only significantly worse than end of double-blind treatment for the first week. During the second week, the mean was higher but not above 95% confidence limits.	Significantly better for at least 2 weeks after discontinuation	No	No
DORA—Daridorexant	Average worse but not tested statistically	Significantly improved over baseline for at least 7 days after discontinuation	No	No
Ramelteon	Yes	Significantly improved over baseline for at least 7 nights	No	No
Doxepin	Means worse but not tested statistically	Improved over baseline for at least 2 nights	No	No
Trazodone	No	Yes	Yes	Nausea, sweating, irritability, agitation, dizziness, sensory disturbances, tremor, anxiety, confusion, headache, lethargy, hypomania, and seizures, among others
Mirtazapine	Yes	Yes	No	Dizziness, sensory disturbances, agitation, anxiety, fatigue, confusion, headache, tremor, nausea, vomiting, and sweating, among others
TCAs	Yes	Yes	No	Nausea, headache, malaise, irritability, and restlessness, among others
Quetiapine	No	No	Yes	Nausea, vomiting, agitation, restlessness, diaphoresis, irritability, anxiety, tachycardia, hypertension, and dizziness, among others

**Table 2:** Consensus recommendation for switching insomnia medications, both within-class and to a new drug class.

Initial Drug Class/Group	Consensus Recommendation for Different Class Switching	Grading of Evidence	Consensus Recommendation for Within-Class Switching	Grading of Evidence
BZDs	Slow taper method/cross taper	B /C	Direct switch	B
Zolpidem	Taper and then wait 1–2 days	B	Taper and then wait 1–2 days	B
Zaleplon	Direct switch	B	Direct switch	B
Eszopiclone	Taper and then wait 1–2 days	B	Taper and then wait 1–2 days	B
Suvorexant	Direct switch	B	Direct switch	B
Lemborexant	Direct switch	B	Direct switch	B
Daridorexant	Direct switch	B	Direct switch	B
Ramelteon	Direct switch	B	N/A	
Doxepin 3–6 mg	Direct switch	B	N/A	
Trazodone	Slow taper method /cross taper	D	Not recommended	E
Mirtazapine	Slow taper method /cross taper	E	Not recommended	E
TCAs	Slow taper method /cross taper	D	Not recommended	E
Quetiapine	Slow taper method /cross taper	D	Not recommended	E

Grading of evidence B—evidence obtained from either meta-analysis, including at least 1 small RCT, or from at least 1 well-designed large RCT, C—evidence obtained from well-designed cohort or case-controlled studies, D—evidence obtained from case series, case reports, or flawed clinical trials, and E—opinions of respected authorities based on clinical experience, descriptive studies, or reports of expert committees. Definition of the consensus-based switching methods are as follows: slow taper: gradual dose reduction of insomnia drug, with lowering by increments every few days, usually over a period of 4 weeks, with the goal of discontinuing the medication. This process’s duration and success depend on drug dosage, pharmacological properties, and subject response to the decreased dose. Cross taper: The first insomnia drug dose is reduced while a new insomnia medication is introduced at a low dose and gradually increased. This can only be safely done with medications that have no interaction. Taper and wait 1–2 days: Similar to the slow taper method of gradually decreasing the dose until discontinuation, followed by a withholding period of 1–2 days before any new insomnia medication is started. This can be due to the insomnia treatment having a longer half-life and needing time to be cleared from the system prior to initiating new therapies. Direct switch: The first insomnia drug is stopped, and a new insomnia drug is commenced the next day at the usual therapeutic dose. There can be a considerable risk of withdrawal symptoms and drug interactions.

## Data Availability

Not applicable.
